# Towards an Accurate Real-Time Digital Elevation Model Using Various GNSS Techniques

**DOI:** 10.3390/s24248147

**Published:** 2024-12-20

**Authors:** Mohamed Abdelazeem, Amgad Abazeed, Hussain A. Kamal, Mudathir O. A. Mohamed

**Affiliations:** 1 Civil Engineering Department, College of Engineering in Al-Kharj, Prince Sattam Bin Abdulaziz University, Al-Kharj 11942, Saudi Arabia; 2 Civil Engineering Department, Faculty of Engineering, Aswan University, Aswan 81542, Egypt; hussainahmed@eng.aswu.edu.eg; 3 Construction and Building Engineering Department, College of Engineering and Technology, Arab Academy for Science, Technology and Maritime Transport, Aswan 81544, Egypt; eng.amgad@aast.edu; 4 Engineering Surveying Department, College of Engineering Sciences, Omdurman Islamic University, Khartoum 11111, Sudan; mudathiro@oiu.edu.sd

**Keywords:** real-time PPP, real-time kinematic (RTK), CNES, digital elevation model (DEM), QGIS

## Abstract

The objective of our research is to produce a digital elevation model (DEM) in a real-time domain. For this purpose, GNSS measurements are obtained from a kinematic trajectory in a clear location in New Aswan City, Egypt. Different real-time processing solutions are employed, including real-time precise point positioning (RT-PPP) and real-time kinematics (RTK); additionally, the widely used post-processed precise point positioning (PPP) processing scenario is used. Thereafter, the acquired positioning estimates are compared with the traditional kinematic differential GNSS solution counterparts. To achieve the RT-PPP mode, the instantaneous products from the Centre National d’Etudes Spatiales (CNES) are utilized. Our proposed models are validated for both kinematic positioning and DEM accuracies. For kinematic positioning accuracy validation, the findings indicate that the three-dimensional position is about 0.480 m, 0.101 m, and 0.628 for RT-PPP, RTK, and PPP solutions, respectively. Furthermore, the DEM accuracy investigation shows that the produced DEMs have accuracies within 0.249 m, 0.005 m, and 0.264 m for RT-PPP, RTK, and PPP solutions, respectively.

## 1. Introduction

The digital elevation model (DEM) is the digital illustration of Earth’s topography; therefore, it is crucial for many applications, including surveying, cartography, urban planning, geology, and hydrology. The accuracy of the produced DEM is based on the properties of the input data, which are horizontal and vertical accuracy, distribution, and density, as well as the interpolation technique.

A number of techniques are applied in order to create the digital elevation model, for example, the unmanned aerial system (UAS) technique [1,2,3,4]. Additionally, light detection and ranging (LiDAR) is used in DEM creation [5,6,7,8]. The multi-sensors data fusion technique can also be utilized in DEM production [9,10,11]. Moreover, there are many freely available digital elevation model sources, including the advanced spaceborne thermal emission and reflection radiometer (ASTER) global digital elevation model (GDEM) [12], the advanced land observing satellite (ALOS) world 3D-30 m (AW3D30) [13], the shuttle radar topography mission (SRTM) [14], and TanDEM-X [15]. The accuracy of these open-source DEMs has been examined by many researchers in different engineering applications, e.g., [16,17,18,19,20,21,22,23].

The accuracy of the aforementioned DEM generation techniques is degraded in the denied areas; therefore, global positioning satellite systems (GNSS) are utilized in DEM creation in both open-sky and denied areas. Different GNSS survey techniques can be used in DEM generation, including precise point positioning (PPP), post-processed kinematics (PPK), real-time kinematics (RTK), and network real-time kinematics (NRTK). The accuracy of the GNSS-derived DEM has been explored by a number of researchers, e.g., [24,25,26]. Wani and Nagai [26] developed a DEM using multi-GNSS observations in an urban area, and the collected observations were then processed in PPK mode. The results indicated that the generated DEM’s accuracy was at the decimeter level.

Furthermore, GNSS surveys have been used for the validation of the DEM’s accuracy, particularly for the open-source DEMs, e.g., [27,28,29,30,31]. For instance, the precision of the TanDEM-X DEM has been validated with respect to the kinematic GNSS PPP solution [27]; the validation was carried out along a total distance of 25,000 km. It was found that the root mean square error (RMSE) for the TanDEM-X compared with GNSS was about 1.125 m. Additionally, Liu et al. [29] examined the vertical accuracy of AW3D, ASTER, STRM, and TanDEM-X DEMs using global positioning system (GPS) control points over China, and the DEM accuracies were explored in both longitude and latitude domains. It was shown that the TanDEM-X DEM’s accuracy had superiority over the other DEMs; in addition, the height accuracy in high latitudes was superior to that in low latitudes, and no obvious differences were noted with respect to longitude.

Our present study’s motivation is to create a digital elevation model in a real-time mode. The GNSS kinematic path dataset is collected; hence, various processing models are developed, including real-time precise point positioning (RT-PPP), RTK, and post-processed PPP. To correct the satellite orbit and clock errors, Centre National d’Etudes Spatiales (CNES) products are applied to the RT-PPP solution, whereas the broadcast ephemeris and the final International GNSS Service (IGS) products are utilized for both the RTK and PPP solutions, respectively. Moreover, the differential solution is used as a reference for both kinematic positioning and DEM verifications.

## 2. Kinematic PPP Mathematical Model

The widely used dual-frequency ionosphere-free GNSS kinematic PPP processing model can take the following formula [32]:

(1) $$P_{IF}=\rho_{r}^{s}+c\left(dt_{r}+{dt}^{s}\right)-c\left(b_{r,IF}+b_{IF}^{s}\right)+T_{r}^{s}+\varepsilon_{p,IF}^{s}$$

(2) $$\Phi_{IF}=\rho_{r}^{s}+c\left(dt_{r}+{dt}^{s}\right)-c\left(\delta_{r,IF}+\delta_{IF}^{s}\right)+T_{r}^{s}+{\lambda N}_{IF}^{s}+\varepsilon_{\Phi ,IF}^{s}$$

where both $P_{IF}$ and $\Phi_{IF}$ represent the ionosphere-free code and carrier combinations, respectively; both s and r denote the GNSS satellite and receiver, respectively; the light’s speed in vacuum is c; $dt_{r}$ and ${dt}^{s}$ are the errors in the receiver and satellite, respectively; $b_{r,\;IF}$ and $\delta_{r,\;IF}$ represent the receiver ionosphere-free differential code and phase biases, respectively; $b_{IF}^{s}$ and $\delta_{IF}^{s}$ refer to the satellite ionosphere-free differential code and phase biases, respectively; $T_{r}^{s}$ is the tropospheric error; λ is the carrier phase’s wavelength; $N_{IF}^{s}$ corresponds to the real-value ambiguity; $\varepsilon_{p,IF}^{s}$ and $\varepsilon_{\Phi ,IF}^{s}$ indicate the measurements noise for both the code and carrier.

For the code observation equation, the differential code bias (DCB) parameters are grouped into both receiver and satellite clocks. The satellite’s clock is corrected using the CNES products, which are the real-time archived products (i.e., CNT) for the RT-PPP model; on the other hand, the IGS products are used for the PPP model. Consequently, the PPP mathematical model can be reformulated as given below:

(3) $$P_{IF}=\rho_{r}^{s}+c{dt}_{r}^{\sim }-c{dt}^{s,corr}+T_{r}^{s}+\varepsilon_{p,IF}^{s}$$

(4) $$\Phi_{IF}=\rho_{r}^{s}+c{dt}_{r}^{\sim }-c{dt}^{s,corr}+T_{r}^{s}+{\tilde{N_{IF}}}^{s}+\varepsilon_{\Phi ,IF}^{s}$$

where ${dt}_{r}^{\sim }$ corresponds to the clock parameter, which is the sum of the clock error and DCB ($c{dt}_{r}^{\sim }=c(dt_{r}+b_{r,IF})$); ${dt}^{s,corr}$ is the adjusted satellite clock parameter; ${\tilde{N_{IF}}}^{s}$ is the ambiguity factor, which can be determined as follows:

(5) $${\tilde{N_{IF}}}^{s}={\lambda N}_{IF}^{s}+[(b_{r,IF}+\delta_{r,IF})-(b_{IF}^{s}+\delta_{IF}^{s})]$$

The tropospheric error term is divided into hydrostatic and wet variables; the hydrostatic term can be estimated using such an empirical model; on the other hand, the wet term is considered an estimable parameter. As a result, the vector of the estimable parameters ($\vec{X_{IF}}$) can take the following mathematical expression:

(6) $$\vec{X_{IF}}=\left[∆x,\;\;\;\;\;∆y,\;\;\;\;\;∆z,\;\;\;\;\;\;c{dt}_{r}^{\sim },\;\;\;\;\;T_{w},\;\;\;\;\;{\tilde{N_{IF}}}^{s}\right]$$

where ∆x,  ∆y, and ∆z are the corrections applied to the receiver’s coordinates; $T_{w}$ points to the wet tropospheric delay parameter.

## 3. GNSS Kinematic Trajectory Survey

**Figure 1:**
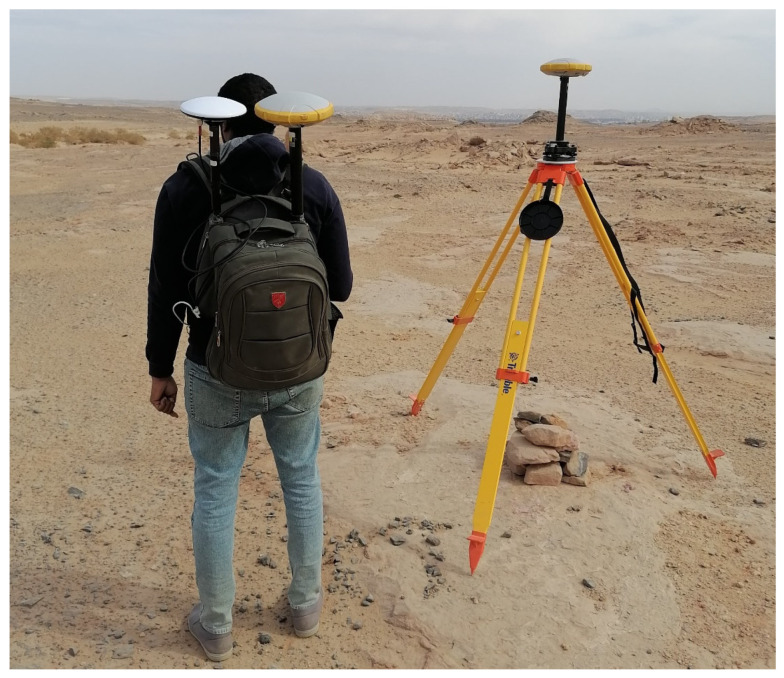
Setup of both base and rover GNSS receivers.

**Figure 2:**
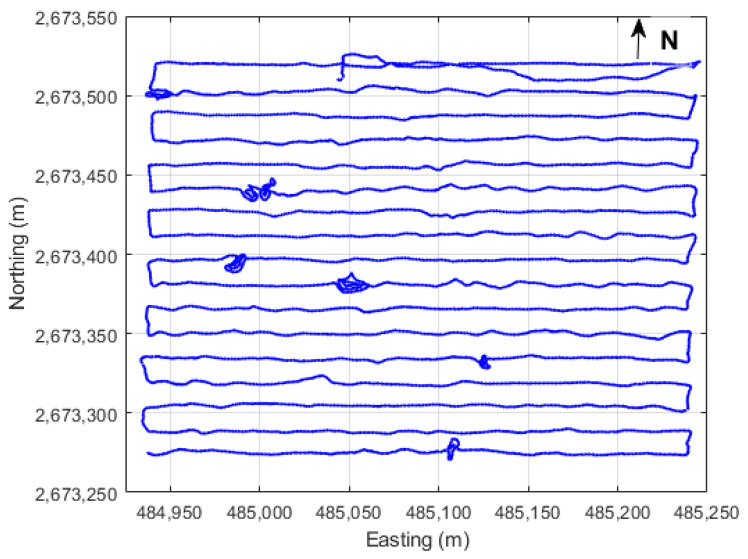
Kinematic trajectory layout (UTM 36N coordinates in meters).

To create our proposed real-time digital elevation model, the kinematic survey is implemented in different steps. Firstly, an open sky land parcel in New Aswan city, Egypt, is selected, the area of which is about 20 acres with a moderate topography. Next, GNSS datasets are collected on the day of year (DOY) 53 in 2024 using the geodetic-grade Trimble R4s module as a base receiver; in addition, another Trimble R4s modules is also used as rover receiver, as shown in Figure 1. It should be mentioned that the kinematic survey is executed using the walking trajectory method, and the kinematic trajectory spans about 2 h. Figure 2 illustrates the land parcel’s boundaries and the walking trajectory pattern. Thereafter, different processing scenarios are employed, including real-time precise point positioning, real-time kinematics, and post-processed precise point positioning solutions.

The Net-Diff GNSS version 1.13 software [33] is used to obtain the aforementioned solutions (i.e., RT-PPP, RTK, and PPP). The dual-frequency ionosphere-free model is used for both the real-time and post-processed PPP solutions; moreover, the Saastamoinen model [34], along with both the wet and dry global mapping functions (GMF) [35], are utilized for tropospheric delay terms. To account for the satellite and clock orbit corrections, both the real-time CNES [36] and the IGS-final [37] products are used for the RT-PPP and PPP solutions, respectively; on the other hand, the multi-GNSS broadcast ephemeris (i.e., BRDM) [38] are used for the RTK solution. The time interval is 1 s for the three solutions; moreover, the Kalman filter is used for parameter estimation. To obtain a reference solution for the three processing solutions, the GNSS measurements from both the base and rover receivers are processed, involving the traditional kinematic differential GNSS solution, which is also provided within the Net_Diff version 1.13 software. The processing parameters for each solution are outlined in Table 1. It should be stated that the extracted coordinates from the four solutions belong to the WGS84/UTM 36N projected coordinate system.

**Figure 3:**
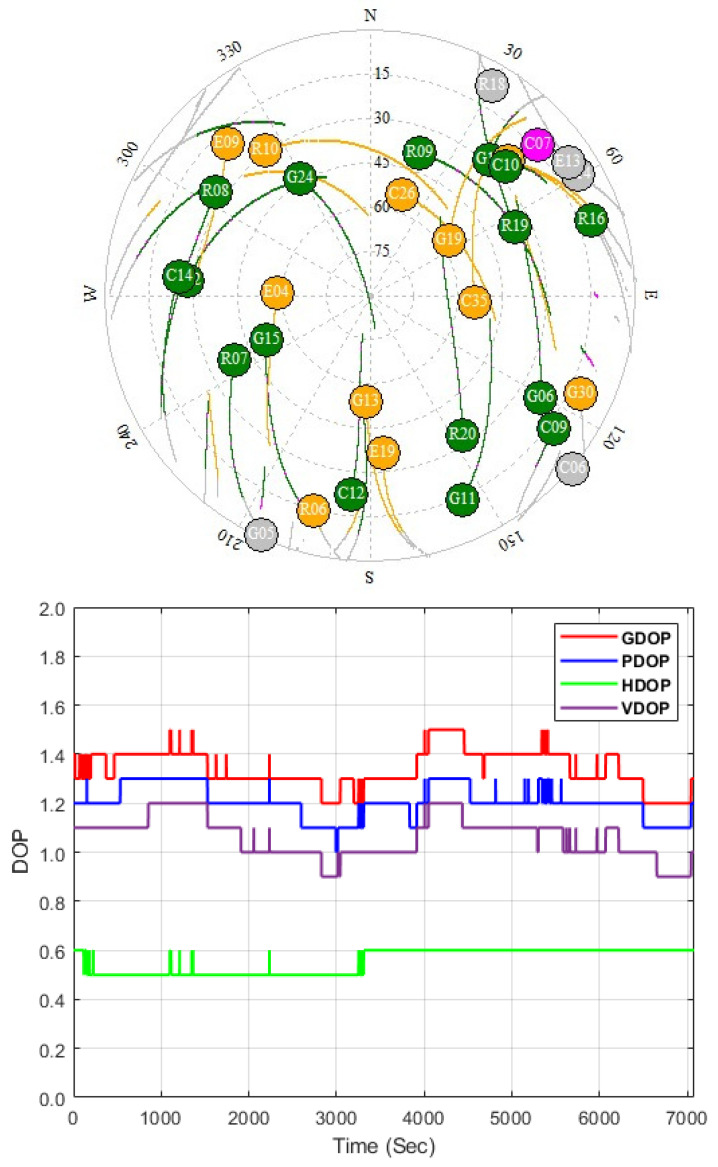
GNSS satellite visibility (**up**) and DOPs (**bottom**) over the studied area.

Both sky visibility and satellite geometry are examined over the selected area during the experimental period because they are crucial parameters for precise positioning; therefore, the dilution of precision (DOP) values is computed. Figure 3 depicts the geometric dilution of precision (GDOP), position dilution of precision (PDOP), horizontal dilution of precision (HDOP), vertical dilution of precision (VDOP), and the satellite distribution. It is clearly seen that the number of the visible GNSS satellites ranges from 19 to 24 satellites, and the DOP values vary between 0.5 and 1.5 during the kinematic survey period; moreover, the PDOP differs smoothly from 1 to 1.3 during the observation time.

## 4. Results and Analysis

**Figure 4:**
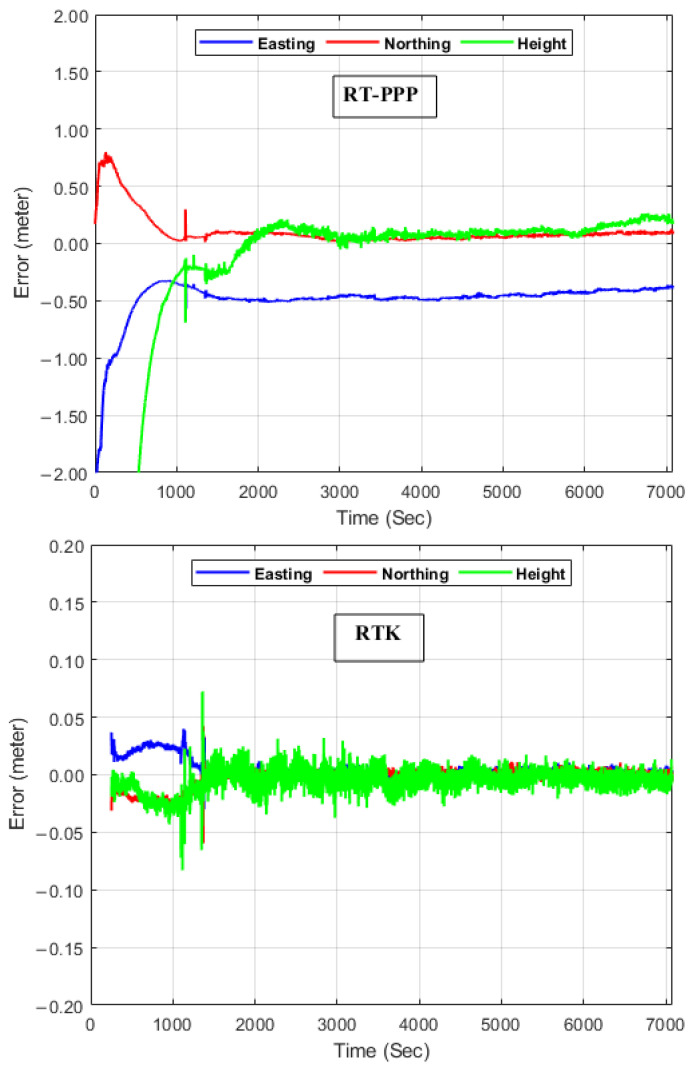

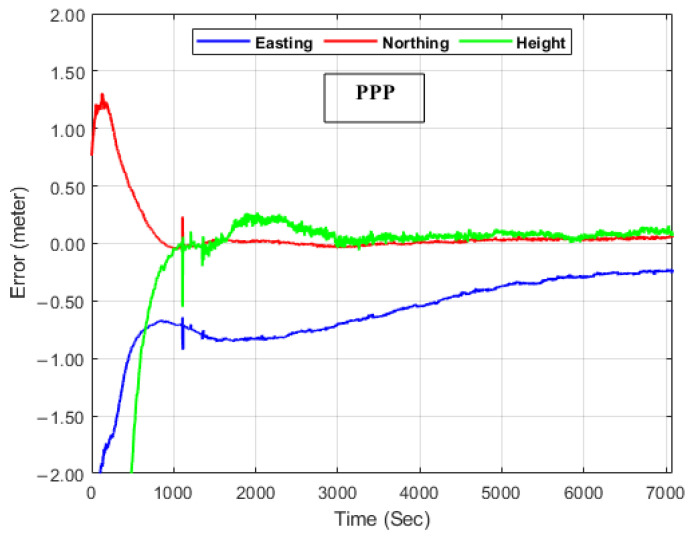
Positioning errors for the RT-PPP, RTK, and PPP solutions.

To explore the accuracy of our produced digital elevation models, the collected kinematic GNSS datasets were processed utilizing various GNSS processing models, which were RT-PPP, RTK, and PPP. The positioning accuracy of the three proposed GNSS solutions was investigated using the kinematic differential GNSS solution as a reference solution. Thereafter, the kinematic PPP positioning errors for the three processing models in easting, northing, and height components were computed and are illustrated in Figure 4. It can be seen that both the RT-PPP and PPP models converge after about 16 min; furthermore, both northing and height directions converge to reach the centimeter level for the RT-PPP and PPP solutions. On the contrary, the easting direction converges to the decimeter level for the two solutions. This can be attributed to the satellite distribution. For the RTK solution, it converges faster than the PPP solutions; additionally, significant improvements in the three components positioning accuracy are attained to reach the few centimeters level. It can also be noted that there is no solution for the first five minutes approximately; this could be due to the fact that the broadcast ephemeris file does not contain satellite positions.

**Figure 5:**
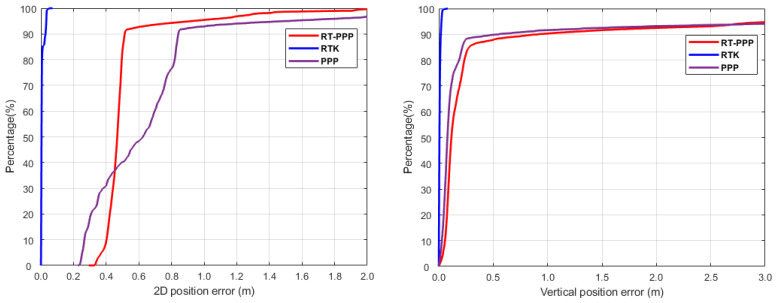
CDF of the horizontal and vertical positions for the three solutions.

To further evaluate the kinematic positioning accuracy, the cumulative distribution function (CDF) of the horizontal and vertical positioning components were computed for the three proposed processing models (Figure 5). It was obvious that about 90% of the 2D positioning errors were less than 0.5 m and 0.8 m for the RT-PPP and PPP models, respectively, whereas about 90% of the height positioning errors were less than 0.5 m for the two solutions. On the other hand, for the RTK solution, about 90% of the positioning errors were less than 0.10 m for both horizontal and height directions.

For better assessment of the kinematic GNSS’s positioning accuracy, statistical parameters, including mean and RMSE for the positioning estimates, were computed; thereafter, these parameters are summarized in Table 2. It can be clearly seen that the RTK solution has significant superiority to the other PPP solutions, with RMSE values of less than 1 decimeter in both horizontal and vertical components; on the contrary, the accuracy of both the PPP and RT-PPP solutions is within the decimeter level with outperformance for the RT-PPP solution.

**Figure 6:**
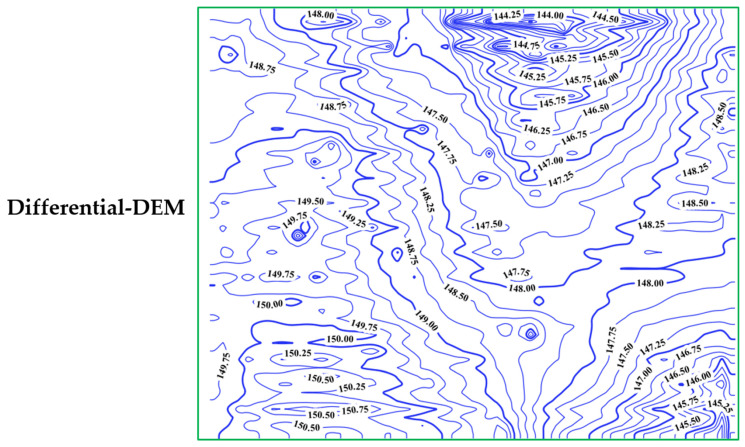

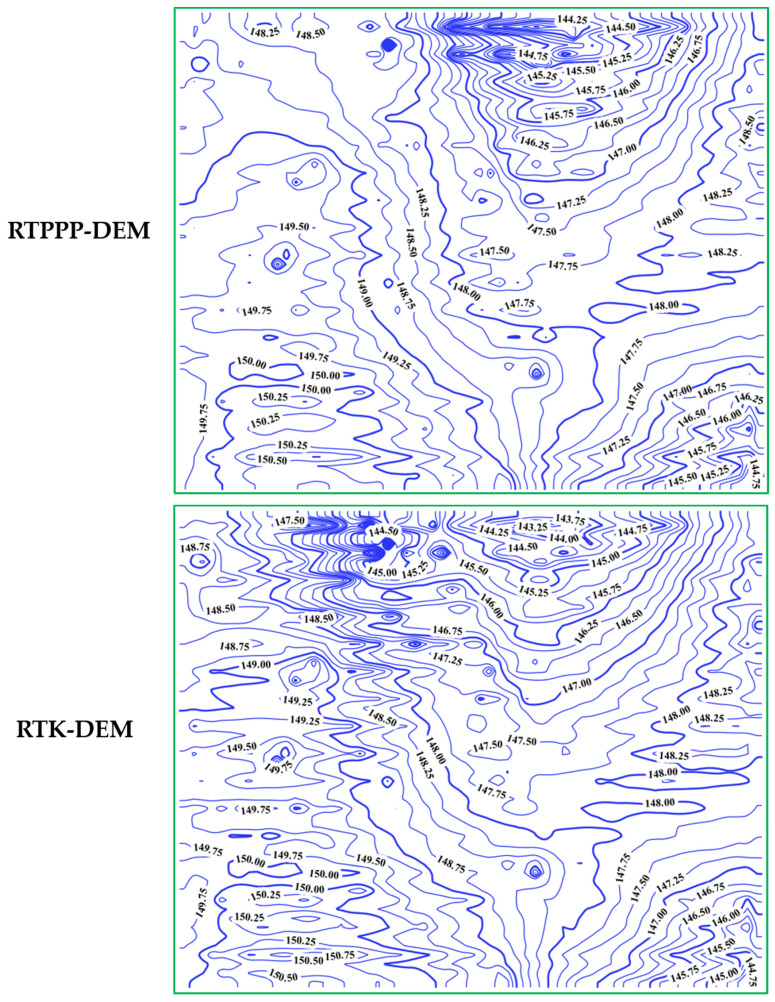

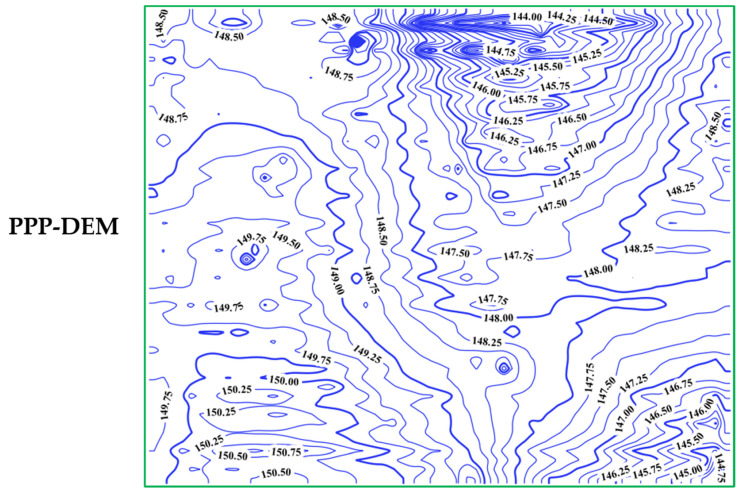
Produced DEMs from differential, RT-PPP, RTK, and PPP.

Another main objective of our paper is to investigate the DEM’s accuracy; therefore, the digital elevation model was derived from the differential, RT-PPP, RTK, and PPP solutions. The resulting ellipsoidal heights from the aforementioned solutions were converted into orthometric heights to be convenient for the DEM applications, which can be estimated using the following formula [32]:

(7) h=H+n

where h is the ellipsoidal height; H refers to the orthometric height; n is the geoid undulation. In our research, the EGM2008 geoid model [39] was utilized to determine the orthometric height; additionally, the inverse distance weighted (IDW) interpolation method was used to produce the DEM for each solution utilizing the QGIS version 3.34 software [40]. The produced DEM had a 5 m × 5 m spatial resolution; in other words, the height value was estimated as a 5 m × 5 m grid with a total number of 3276 points. Figure 6 shows the derived contour map from the differential, RT-PPP, RTK, and PPP solutions, respectively. It can be clearly seen that the observed parcel has its highest elevation at the south-west corner, and the elevation gradually decreases in all other directions; in addition, it has significant elevation variations in both the north-east and south-east directions.

**Figure 7:**
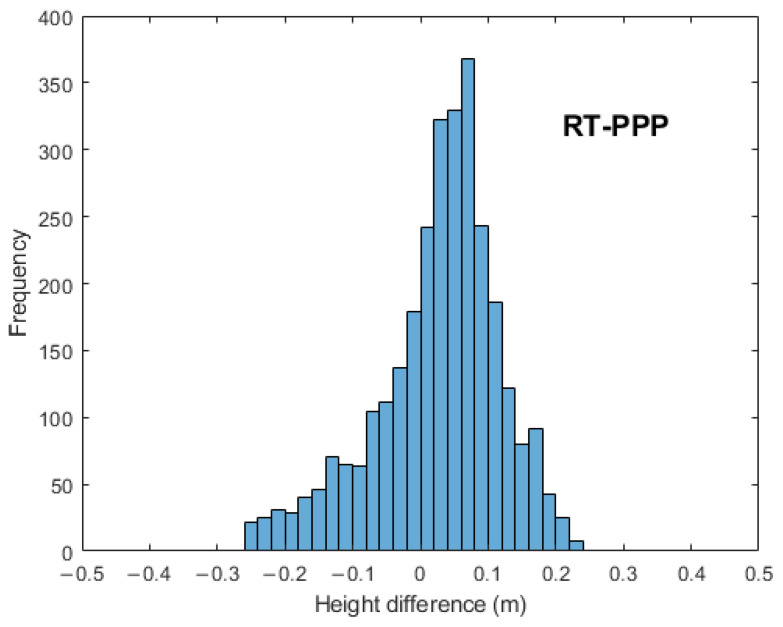

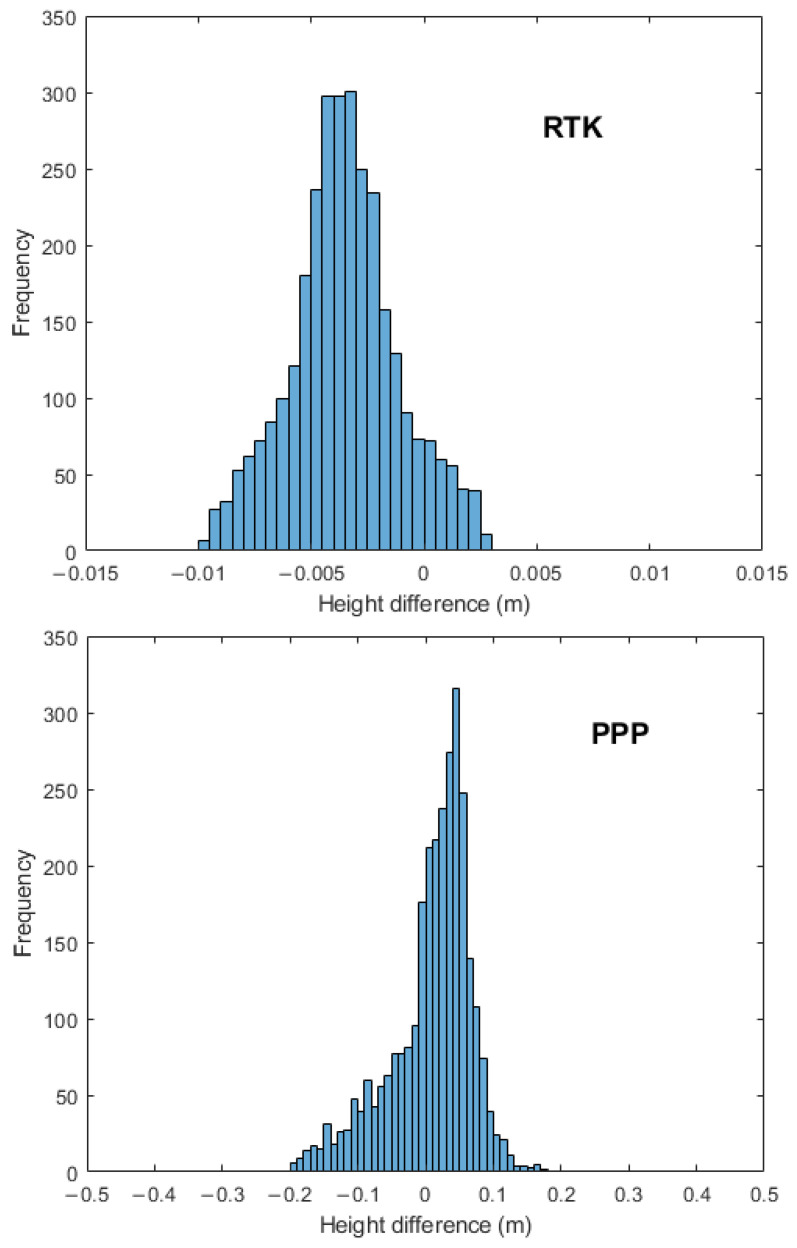
Histogram of height differences for RT-PPP, RTK, and PPP.

To evaluate the accuracy of the created digital elevation models, the orthometric height difference was computed by subtracting the differential height from its corresponding RT-PPP, RTK, and PPP height at each grid point; the histograms for the elevation differences are plotted in Figure 7.

It was clear that the height variations illustrated a uniform distribution, and they ranged from −0.25 m to 0.25 m and from −0.20 m to 0.20 m for both the RT-PPP and PPP models, respectively; on the other hand, for the RTK-derived DEM, the height difference distributions ranged from −0.01 m to 0.0025 m, which indicates its superiority compared with the other two DEMs.

**Figure 8:**
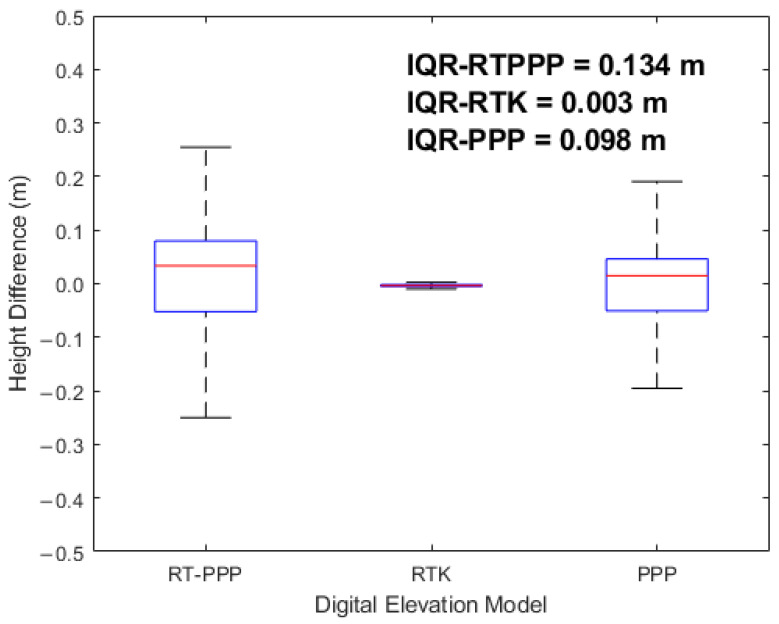
Box plot of the height differences.

To better visualize and analyze the height differences for each of our proposed digital elevation models, a box plot was utilized to depict the orthometric height discrepancies (Figure 8). It was clear that the elevation discrepancies were widely distributed for both the RT-PPP and PPP digital elevation models, while they were ultra-narrowly distributed for the RTK digital elevation model; consequently, the interquartile range (IQR) values were 0.134 m, 0.003 m, and 0.098 m for the RT-PPP, RTK, and PPP DEM solutions, respectively.

A comprehensive statistical analysis for our generated digital elevation models was performed to further explore their accuracy, as given in Table 3, which included mean bias error (MBE), RMSE, standard deviation (STD), and probable error (STD_50%_); moreover, the confidence levels at 90%, 95%, and 99% intervals were also computed (i.e., STD_90%_, STD_95%_, and STD_95%_, respectively). The aforementioned statistical parameters were computed as given below:

(8) $$∆H=H_{Differntial\;}-H_{solution\;}$$

(9) $$MBE=\frac{1}{n}\sum_{i=1}^{n}{∆H}_{i}$$

(10) $$RMSE=\sqrt{\frac{1}{n}\sum_{i=1}^{n}{∆H}_{i}^{2}}$$

(11) $$STD=\sqrt{\frac{1}{n-1}\sum_{i=1}^{n}{\left({∆H}_{i}-MBE\right)}^{2}}$$

(12) $${STD}_{50\%}=0.675*STD\;{STD}_{90\%}=1.645*STD\;{STD}_{95\%}=1.960*STD\;{STD}_{99\%}=3.00*STD$$

where ∆H is the orthometric height difference; $H_{Differntial\;}$ refers to the derived orthometric height from the differential solution; $H_{solution\;}$ indicates the derived orthometric height from the other solutions (i.e., RT-PPP, RTK, and PPP); MBE is the mean bias error; i denotes the grid point; n is the total number of grid points; RMSE and STD refer to the root mean square error and standard deviation, respectively; ${STD}_{50\%},$ ${STD}_{90\%},\;{STD}_{95\%}$, and ${STD}_{99\%}$ are 50%, 90%, 95%, and 99% errors, respectively.

It was obviously shown that the RMSE values were within a few decimeters for both the RT-PPP and PPP-derived DEMs, with slight superiority to the RT-PPP one; moreover, the RTK-derived DEM had significant superiority to the other DEMs, with a 0.005 m RMSE value. In addition, the standard deviation estimates were 0.247 m and 0.257 m for the DEMs produced from both the RT-PPP and PPP solutions, respectively; on the contrary, it was within the millimeter level for the DEM produced from the RTK solution. It could also be seen that the probable errors were about ±0.167, ±0.002, and ±0.174 m for the RT-PPP, RTK, and PPP-generated DEMs, respectively; furthermore, the elevation error at the 90% confidence interval was about ±0.406 m, ±0.005 m, and ±0.423 m for the RT-PPP, RTK, and PPP-derived DEMs, respectively.

## 5. Discussion

The digital elevation model is an important tool for earth topography-related applications, such as surveying, planning, and hydrology applications. For this purpose, a number of techniques are used to produce digital elevation models, including GNSS, LiDAR, and remote sensing. The GNSS technique is widely utilized for DEM creation, particularly the traditional differential GNSS method; however, the limitation of the differential GNSS technique is that it is applied in the post-processing domain. Therefore, we propose creating a digital elevation model in the real-time domain, utilizing real-time precise point positioning and real-time kinematic techniques. For this reason, a kinematic GNSS survey was carried out in a moderate topography open-sky land parcel, and the collected observations were then processed using different processing models, including RT-PPP, PPP, and RTK; moreover, the differential solution was used to provide a reference solution. Hence, the digital elevation model for the selected land parcel was created using the four aforementioned solutions.

Our developed DEMs are valid for earthwork volume computation because it is crucial for many civil engineering applications, such as building constructions, roads, and hydrological projects. Table 4 summarizes the estimated earthworks volume for our produced differential, RT-PPP, RTK, and PPP digital elevation models, which is computed above the given graded elevations utilizing the QGIS version 3.34 software. As can be seen, as the graded elevation increases, the earthworks volumes decrease; in addition, the RTK’s volume values are closer to the differential counterparts compared to the RT-PPP and PPP counterparts.

**Figure 9:**
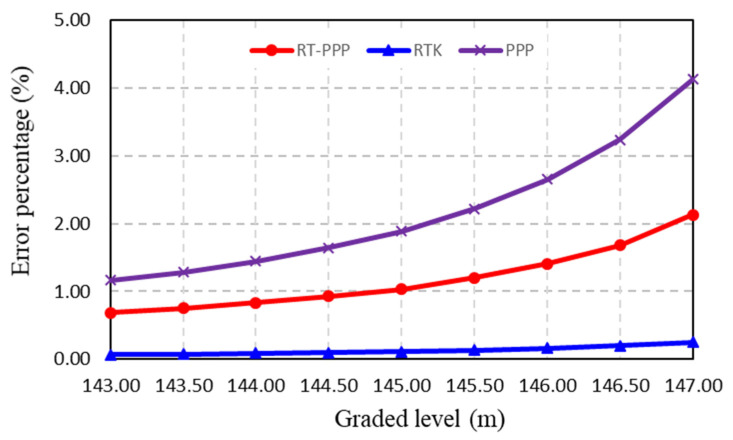
Error percentages for earthworks volume calculations.

To better evaluate the accuracy of the earthworks volume calculations, the differences in earthwork volumes between the differential DEM and the other DEMs were calculated at the given graded elevations; then, the error percentages for these differences were determined at each graded elevation, as shown in Figure 9. It can be clearly seen that the RTK DEM has the lowest error percentage, whereas the RT-PPP DEM is superior to the PPP DEM; moreover, the highest graded elevation is the maximum error percentage. For instance, at 143.00 graded elevation, the error percentages in the earthwork volume calculation are about 0.69%, 0.07%, and 1.16% for the RT-PPP, RTK, and PPP DEMs, respectively; on the other hand, they are about 2.13%, 0.25%, and 4.13% at 147.00 graded elevation for the RT-PPP, RTK, and PPP DEMs, respectively.

Based on the results of both the kinematic positioning and DEM validations, the following conclusions can be drawn:

- The three-dimensional kinematic positioning accuracy is within one decimeter level for the RTK scenario, whereas it is about five and six decimeters for the RT-PPP and PPP scenarios, respectively;
- For DEM validation, the RTK-derived DEM has superiority in comparison to both the RT-PPP and PPP counterparts;
- The created DEM from the RT-PPP solution outperforms the PPP DEM for both the elevation’s accuracy and earthworks volume validations;
- It can be said that with one receiver, an accurate, real-time, cost-effective digital elevation model can be produced; thus, this produced DEM can be used in many civil engineering applications;
- The RTK solution produces a highly accurate DEM; however, its limitation is the need for two receivers. On the other hand, RT-PPP is an affordable solution because only one receiver is used.

## 6. Conclusions

In our study, a real-time digital elevation model was proposed utilizing RT-PPP and RTK scenarios; for this reason, a GNSS kinematic survey campaign was carried out in an open-sky land parcel. After that, the collected GNSS observations were processed in RT-PPP and RTK modes; both the PPP and differential modes were also involved. The differential GNSS solution was utilized as a reference solution. To account for the RT-PPP scenario, the CNES products were used. Our proposed DEMs were studied for kinematic position estimates, height differences, and earthworks volume determination. The findings revealed that the RTK solution was superior to the others in terms of 3D positioning accuracy, DEM creation, and earthwork volumes, while the RT-PPP solution was better than the PPP solution. As a consequence, the derived RT-PPP DEM is an affordable real-time DEM that can be used in a number of engineering applications, such as civil engineering and hydrology and geology applications.

## Figures and Tables

**Table 1:** Processing parameters for each utilized solution.

Parameter	Solution			
	RT-PPP	RTK	PPP	Differential
System	GNSS	GNSS	GNSS	GNSS
Mathematical model	Undifferenced	Differenced	Undifferenced	Differenced
Tropospheric model	Saastamoinen model + Global mapping function			
Sampling rate	1 Hz			
Mask angle	10°			
Orbits and clocks	CNES	BRDM	IGS-final	IGS-final
Parameter estimation	Kalman filter			

**Table 2:** The kinematic positions’ statistical analysis (in meters).

Parameter	RT-PPP			RTK			PPP		
	2D	H	3D	2D	H	3D	2D	H	3D
Mean	0.454	0.016	0.454	0.015	−0.083	0.084	0.548	0.048	0.550
RMSE	0.456	0.151	0.480	0.071	0.072	0.101	0.588	0.220	0.628

**Table 3:** Statistical parameters for the elevation differences.

DEM Model	Statistical Parameters (m)						
	MBE	RMSE	STD	STD_50%_	STD_90%_	STD_95%_	STD_99%_
RT-PPP	−0.035	0.249	0.247	0.167	0.406	0.484	0.741
RTK	−0.004	0.005	0.003	0.002	0.005	0.006	0.009
PPP	−0.060	0.264	0.257	0.174	0.423	0.504	0.772

**Table 4:** Earthworks volume at various graded levels for the proposed DEMs.

Graded Elevation (m)	Differential (m^3^)	RT-PPP (m^3^)	RTK (m^3^)	PPP (m^3^)
143.00	411,201	414,022	411,482	415,974
143.50	371,303	374,095	371,585	376,075
144.00	331,504	334,271	331,786	336,285
144.50	291,926	294,638	292,210	296,722
145.00	252,858	255,472	253,144	257,637
145.50	214,436	217,011	214,721	219,200
146.00	177,088	179,580	177,371	181,781
146.50	141,095	143,471	141,375	145,658
147.00	106,795	109,066	107,065	111,206

## Data Availability

The data presented in this study are available on request from the authors.
